# Discovery of New Compounds Active against *Plasmodium falciparum* by High Throughput Screening of Microbial Natural Products

**DOI:** 10.1371/journal.pone.0145812

**Published:** 2016-01-06

**Authors:** Guiomar Pérez-Moreno, Juan Cantizani, Paula Sánchez-Carrasco, Luis Miguel Ruiz-Pérez, Jesús Martín, Noureddine el Aouad, Ignacio Pérez-Victoria, José Rubén Tormo, Víctor González-Menendez, Ignacio González, Nuria de Pedro, Fernando Reyes, Olga Genilloud, Francisca Vicente, Dolores González-Pacanowska

**Affiliations:** 1 Instituto de Parasitología y Biomedicina “López-Neyra”, Consejo Superior de Investigaciones Científicas, Parque Tecnológico de Ciencias de la Salud, Avenida del Conocimiento, s/n, 18016-Armilla (Granada), Spain; 2 Fundación MEDINA, Parque Tecnológico de Ciencias de la Salud, Avenida del Conocimiento, 34.18016-Armilla (Granada), Spain; Monash University, AUSTRALIA

## Abstract

Due to the low structural diversity within the set of antimalarial drugs currently available in the clinic and the increasing number of cases of resistance, there is an urgent need to find new compounds with novel modes of action to treat the disease. Microbial natural products are characterized by their large diversity provided in terms of the chemical complexity of the compounds and the novelty of structures. Microbial natural products extracts have been underexplored in the search for new antiparasitic drugs and even more so in the discovery of new antimalarials. Our objective was to find new druggable natural products with antimalarial properties from the MEDINA natural products collection, one of the largest natural product libraries harboring more than 130,000 microbial extracts. In this work, we describe the optimization process and the results of a phenotypic high throughput screen (HTS) based on measurements of *Plasmodium* lactate dehydrogenase. A subset of more than 20,000 extracts from the MEDINA microbial products collection has been explored, leading to the discovery of 3 new compounds with antimalarial activity. In addition, we report on the novel antiplasmodial activity of 4 previously described natural products.

## Introduction

Malaria is widespread in tropical and subtropical regions, including parts of America, Asia and Africa. An estimated 3.2 billion people are at the risk of suffering malaria and from one-half to one million deaths were reported in 2014 (*World Malaria Report*. 2014). In 2014, 97 countries and territories had ongoing malaria transmission. Most deaths from malaria are caused by *Plasmodium falciparum*, one of the five species of human infectious malaria parasites. The increasing resistance of *P*. *falciparum* to the available drugs [1] and new efforts to eradicate malaria all drive the need to develop new, effective and affordable antimalarial agents.

Despite the development of new technologies to study resistance acquisition [2–4] and our increasing understanding of *P*. *falciparum* biology, few new drug targets have been clinically validated. At present, there are only four classes of valid antimalarial compounds: quinine or other aminoquinolines, antifolate compounds, artemisinin derivatives, and the hydroxyl napthoquinone atovaquone. This lack of structural diversity denotes a need to explore other sources of structures, and natural products from microorganisms render a unique chemical space for this purpose.

Natural products are one of the most important sources for new chemical scaffolds. They have been largely exploited in the discovery of new drugs, and around 60% of the drugs available nowadays derive directly or indirectly from natural products [5, 6]. Many of the antibiotics or drugs in use such as camptothecin, lovastatin, maytansine, paclitaxel, reserpine and silibinin are all natural products. Some of the first-line malaria treatments currently used are isolated from plants, such as artemisin and quinine. On the other hand, microbial natural products have been underexplored in this field, although they offer great advantages for the potential discovery of novel bioactive products and the possibility of large-scale production. Unfortunately, to date, natural product libraries have not been extensively used in the search for new antimalarials in large-scale campaigns using high throughput screening (HTS) [7, 8].

Drug discovery through HTS allows the large-scale testing of potentially active products, accelerating the identification of molecules for further development. There are several methods for detecting erythrocyte infection and drug susceptibility. However, not all of these assay formats are suitable for HTS due to several factors such as cost, safety, assay stability, equipment availability and quality of data produced. Frequently, methods for HTS technology are based on the measurement of DNA content in strains of malaria parasites using SYBR Green [9], GFP [10], and 4',6'-diamidino-2-phenylindole [11], or in a stably expressed cytoplasmic firefly luciferase parasite strain (3D7-luc) [12, 13]. Nevertheless, since its description [14], the lactate dehydrogenase (LDH) assay has been increasingly used for *Plasmodium* growth determination, due to its robustness and specificity. PfLDH activity measurements, which are proportional to culture parasitaemia, provide specificity through the use of 3-acetylpyridine adenine dinucleotide (APAD) as cofactor, since the human homologue present in red blood cells carries out this reaction at a very slow rate in the presence of this cofactor instead of NADH. In the present work, we have screened more than 20,000 natural extracts from the MEDINA collection against *P*. *falciparum* using the assay based on LDH activity. This is the first time that this screening approach has been applied directly to the study of natural extracts from a high diversity of microorganisms. Using this methodology, we have identified 7 compounds with antimalarial activity. Three are new/novel structures of which two have been previously described as a result of this screening [15, 16] while pepstatin K is reported herein for the first time. Four are known compounds whose antimalarial properties had not been previously reported. All these findings provide an encouraging starting point that supports a renovated interest in discovering and optimizing novel antimalarial compounds from microbial natural products.

## Materials and Methods

No specific permissions were required for the collection of samples in the Vallibierca valley, Huesca, Spain because Spanish legislation does not regulate the access to soils in public areas (since it is neither a National Park nor a private owned land). We confirm that the studies involve only soil samples and these samples do not involve endangered or protected species.

### Reagents

AlbuMAX II, RPMI medium 1640 and SYBR Green I nucleic acid gel stain were purchased from Gibco (Life Technologies, Carlsbad, CA, USA). Gentamicin, hypoxanthine, chloroquine diphosphate salt, sodium l-lactate, APAD, nitrotrezalium blue chloride (NBT) and diaphorase from *Clostridium kluyveri* were obtained from Sigma Aldrich (St. Louis, MO, USA).

### Cultivation of *P*. *falciparum*

Cultures of *P*. *falciparum* strain 3D7 were maintained in fresh type 0 positive (0^+^) human erythrocytes (Centro Regional de Transfusiones Sanguíneas-Biobanco, Granada) suspended at 5% hematocrit in complete medium containing 2% human 0^+^ serum, 0.5% albuMAX II (Gibco), 12.5 μg/mL gentamicin (Sigma Aldrich), 0.2% sodium bicarbonate (Sigma), 150 μM hypoxanthine (Sigma Aldrich), and 10.4 g/L RPMI 1640 (Gibco) pH 7.2. Cultures were incubated at 37°C under a gas mixture of 5% CO_2_, 1% O_2_ and 94% N_2_. For general cultures, parasites were maintained in non-synchronous forms between 0.1% and 8% parasitaemia in 75 mm flasks. Parasite growth was monitored using thin blood smears fixed in 100% methanol and stained for 20–30 min in 10% Giemsa solution.

### Preparation of *P*. *falciparum* cultures for assay

The parasite *P*. *falciparum* 3D7 cultures were synchronized using 5% sorbitol as previously described [17] and 96 h later, the level of parasitaemia was determined by light microscopy counting of a minimum of 500 erythrocytes on a Giemsa-stained thin blood smear. Parasites were noted to be late-ring and early trophozoites. The stock culture was then diluted with complete medium and normal human erythrocytes to a starting 2% hematocrit and 0.25% parasitaemia in 25 μL of volume for 384-well plates (LDH assay) and 5% hematocrit and 0.5% parasitaemia in 90 μL for 96-well plates (fluorescent assay). Both were incubated at 37°C for 72 h and then frozen for at least 24 h.

### LDH assay conditions

The extracts, fractions and pure compounds were evaluated in 384-well plates after 72 h of incubation. Each plate also included positive growth controls, where only medium was added, and negative growth controls with 100 nM of chloroquine. Plates were thawed at room temperature for at least 1 h. To evaluate LDH activity, 70 μL of freshly made reaction mix, containing 143 mM sodium l-lactate, 143 μM APAD, 178.75 μM NBT, 1 U/mL diaphorase, 0.7% Tween 20 and 100 mM Tris-HCl (pH 8.0) were dispensed into plates. Plates were shaken to ensure mixing and absorbance was measured at 650 nm after 10 min of incubation at room temperature. Absorbance was determined with a microplate reader VICTOR2 Wallac spectrofluorometer. The read time for each plate was 3 minutes. Plates were prepared at 3.5 minute intervals and read in a sequential order in the plate reader. This method gives a signal to noise ratio of 10 under the conditions used [18]. With the 384-well plates, integrity of erythrocytes and LDH activity can be inspected visually, allowing for the rapid detection of dispensing errors and interferences by extracts. Yellow wells contain active inhibitors where parasite growth has been abolished and the LDH reaction has been inhibited. The EC_50_ for chloroquine obtained this way was 8.72 ± 0.29 nM (S1 Fig), which was similar to previously reported values [19–21].

### SYBR Green assay conditions

For the fluorescence assay, after 72 h of growth, 100 μL of SYBR Green I in lysis buffer (0.2 μL of SYBR Green I/mL of lysis buffer containing 20 mM Tris-HCl pH 7.5, 5 mM EDTA, 0.008% saponin and 0.08% Triton X-100) was added to each well, and the contents were mixed. After 2 h of incubation in the dark at room temperature, fluorescence was measured with a Spectra MAX GEMINI EM microplate reader (Molecular Devices) with excitation and emission wavelengths of 485 and 530 nm, respectively.

### Absorbance measurement, visual inspection and statistical analyses

The inhibition percentage of each extract was determined by the equation:

$$Percentage\;of\;growth\;inhibition\;=\;\left[1-\left(\frac{A_{well}-A_{neg}}{A_{pos}-A_{neg}}\right)\right]\;x\;100$$

Where A_neg_ is the optical density of the negative control at 650 nm and A_pos_ is the optical density of the positive control at 650 nm. Data were analyzed using the Genedata Screener program, Condoseo module (Genedata AG, Switzerland). An extract was considered to have activity when the percentage of growth inhibition was higher than 70%. The Z’ factor predicts the robustness of an assay by taking into account the mean and standard deviation of both positive and negative assay controls. The robust Z’ factor (RZ’ factor) is based on the Z’ factor, but standard deviations and means are replaced by the robust standard deviations and medians, respectively. In all experiments performed in this work where a minimum of 700 384-well plates have been used, the RZ’ factor obtained was between 0.7–0.8.

### Microbial extracts collection

For the primary screening campaign, a subset of 20,000 microbial extracts from different modules of the MEDINA natural products collection (Table 1) was used. The microbial extracts were obtained from bacterial and fungal strains cultivated in different nutritional conditions and extracted with acetone (1:1) for 1 h in an orbital shaker. Extracts were then centrifuged at 1500 xg for 15 min and the supernatant concentrated to half the volume in the presence or not of a final concentration of 20% DMSO. Extracts were stored at -20°C in 96-well *ABgene* v-bottom plates until needed.

**Table 1:** Characteristics of the collections assayed against *P*. *falciparum*.

	Organism	Solvent	Other characteristics
Module A	Fungi and bacteria (actinomycetes)	100% water, 1x whole broth equivalent (WBE)	Wide microbial diversity and production conditions
Module B	Fungi and bacteria (actinomycetes)	20% DMSO/water, 1x WBE	Enriched module containing selected strains producing bioactive compounds
Module C	Non-filamenting bacteria	20% DMSO/water 4x WBE	Diverse bacterial taxa

### Primary screening and dose-response experiments

All the extracts were first screened against *P*. *falciparum* and those exhibiting over 70% growth inhibition were selected as actives and confirmed in the LDH and SYRB Green assays. The extracts selected from this stage were then tested in a five-point dose-response assay, using as the first titration point the dilution identified as active in the primary screen and then performing four subsequent 2-fold serial dilutions. Extracts that exhibited dose-responses indicative of good potency were selected for de-replication by tandem liquid chromatography mass spectrometry (LC-MS). LC-MS analyses were carried out as described previously [22].

### Bioassay-guided extract fractionation

Extracts with LC-MS profiles suggestive of containing active novel compounds were selected to confirm the activity from a 100 mL regrowth of the producing strain in the same production conditions [22, 23]. Hits containing compounds of interest detected in the previous step were regrowth at 1 L scale. Extraction with acetone and a first chromatographic separation using SP-207ss brominated polystyrenic resin was performed as previously described [16]. Active fractions from this first chromatographic step were subjected to one or several steps of preparative and semi-preparative reversed phase HPLC on a Gilson GX-281 apparatus until a pure compound was obtained. The chromatographic column, solvent system and gradient conditions for each HPLC separation were selected depending on the particular compound of interest contained in each sample.

### Isolation of pepstatins A and K

The compounds were isolated from 1 L growth of *Kitasatospora mediocidica* F-136,264. The producing strain was isolated from a soil sample collected under a *Juniperus communis* tree collected in the Vallibierca valley, Huesca, Spain. A seed culture of strain F-136,264 was prepared in a inoculum medium (soluble starch 20 g/L, dextrose 10 g/L, NZ amine EKC (Sigma) 5 g/L, Difco beef extract 3 g/L, Bacto peptone 5 g/L, yeast extract 5 g/L, and CaCO_3_ 1 g/L, adjusted to pH 7.0 with NaOH before addition of 1g/L CaCO_3_), at 28°C with 220 rpm orbital shaking. A 5% (v/v) of the seed culture was used to inoculate each of the seven 500 mL flasks containing 150 mL of the production medium (corn dextrin 20 g/L, beta cyclo dextrin 10 g/L, tomato paste 20 g/L, Bacto yeast extract 10 g/L, CoCl_2_.6H_2_O 5 mg/L), and the flasks were incubated at 28°C for 7 days in a rotary shaker at 220 rpm and 70% humidity before harvesting. The 1 L culture was extracted with acetone (1 L) under continuous shaking at 220 rpm for 3 h. The mycelium was then separated by centrifugation and the supernatant (ca. 2 L) was concentrated to 1 L under a stream of nitrogen. This solution was loaded (with continuous 1:1 water dilution, discarding the flow-through) on a column packed with SP-207SS reversed phase resin (brominated styrenic polymer, 65 g) previously equilibrated with water. The column was further washed with water (1 L) and afterwards eluted at 8 mL/min on an automatic flash-chromatography system (CombiFlashRf, Teledyne Isco) using a linear gradient from 10% to 100% acetone in water (in 12.5 min) with a final 100% acetone step of 15 min, collecting 9 fractions of 20 mL. LC-MS analysis allowed the identification of pepstatin A [24] and a new member of this family of compounds that we designated as pepstatin K in the bioactive fraction. This fraction was further purified by reversed phase semipreparative HPLC (Agilent Zorbax SB-C8, 9.4 × 250 mm, 7 um; 3.6 mL/min, UV detection at 210 nm) with a linear gradient of water-CH_3_CN of 5% to 100% CH_3_CN over 37 min to yield pure pepstatin A (11 mg) and pepstatin K (8 mg).

## Results

### Optimization of assay in a 384-well format

**Fig 1:**
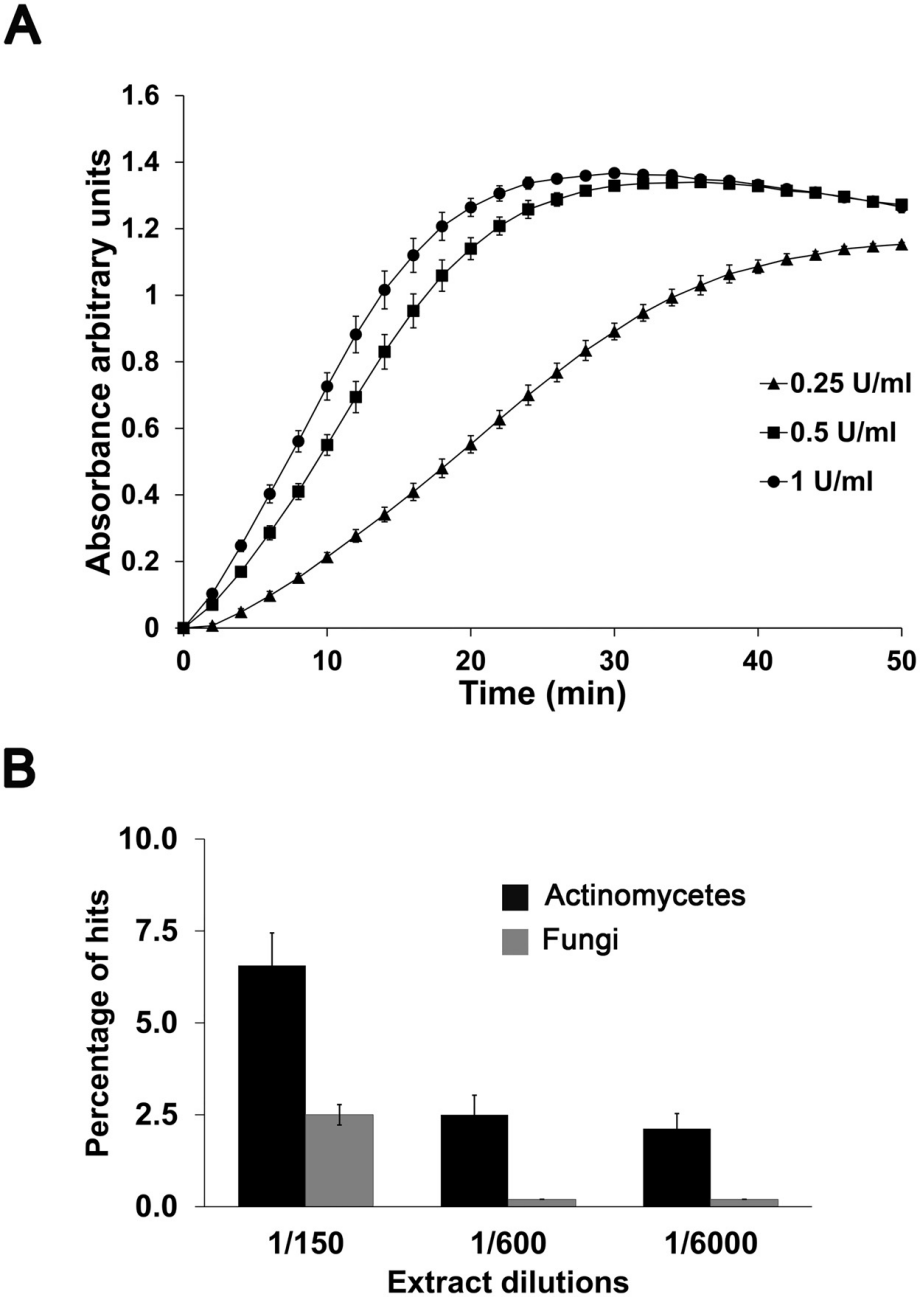
Screening set up. A. Different diaphorase concentrations in the developing buffer were assayed. The absorbance was measured kinetically, taking absorbance data every two minutes verifying that the linearity of the assay comprises the 10 first minutes, time used for the development of plates. B. Percentage of hit rates depending on the dilution applied and the organism origin of the extract. Different dilutions were applied in a pilot sample of module A, comprising both actinomycetes and fungi. Extracts with ≥ 70% parasite growth inhibition were selected as hits.

For the primary screening of a subset of the MEDINA collection, the PfLDH assay was used. This methodology has been previously reported to be adequate for a 384-well plate format in HTS [18], being a robust, sensitive, selective and reproducible assay. We adapted the method to our laboratory conditions and facilities. Firstly, we tried different hematocrits and parasitaemias including those described in the above-mentioned paper. However, while conditions such as 5% haematocrit and 0.5% parasitaemia gave rise to limited linearity of the assay, a 2% haematocrit and 0.25% parasitaemia provided a perfect correlation between growth and signal. In addition, we tested different concentrations of diaphorase ranging from 2.83 U/mL to 0.5 U/mL, not observing differences in the slope of the curve. On the other hand, concentrations below 0.5 U/mL gave lower rates (Fig 1A) and, thus, the final concentration of diaphorase to be used in the screening was established at 1 U/mL.

The SYBR Green protocol was used as a confirmatory counter-assay in a 96-well plate format [25]. This assay quantifies DNA (number of parasites) and thus allows to disregard extracts that interfere or are inhibitors in the LDH assay. Other fluorescent DNA dyes such as YOYO and the mixture of YOYO and SYBR Green, a combination previously used in HTS campaigns [26], were also tested (data not shown).

MEDINA’s extract collection comprises various modules, characterized by the diversity of the microbial strains represented (mainly bacteria, actinomycetes and fungi), the complexity of the natural product extracts (whole broth crude extracts, SPE extracts or fractions) and the different extraction procedures. We defined the sample dilutions to be applied to the different modules of the collection in the assay according to the percentage of positive hits obtained, to achieve a representative number of hits that could be adequately handled in terms of scale up capabilities. In order to adjust the most appropriate assay dilution of the original collection, we tested crude extracts from actinomycetes and from fungi at three different concentrations. The desired hit rate per 384-well plate was approximately 2.5% when applying a cut-off value for positives of 70% growth inhibition. In both cases, the hit rate decreased with increasing dilutions. Extracts from actinomycetes showed a higher hit rate than extracts from fungi and consequently the dilutions set for these extracts were 1/200 and 1/50, respectively (Fig 1B).

Certain modules of the MEDINA extract collection contain 20% of DMSO, a solvent that inhibits *Plasmodium* growth at concentrations as low as 0.001%. To avoid side effects due to DMSO, aliquots of these extracts were previously evaporated, thus eliminating all the DMSO present, and re-dissolved in methanol, a solvent which is less aggressive against *Plasmodium* cultures and does not affect parasite growth even at 1%.

### Screening campaigns, identification of actives and LC-MS de-replication

**Fig 2:**
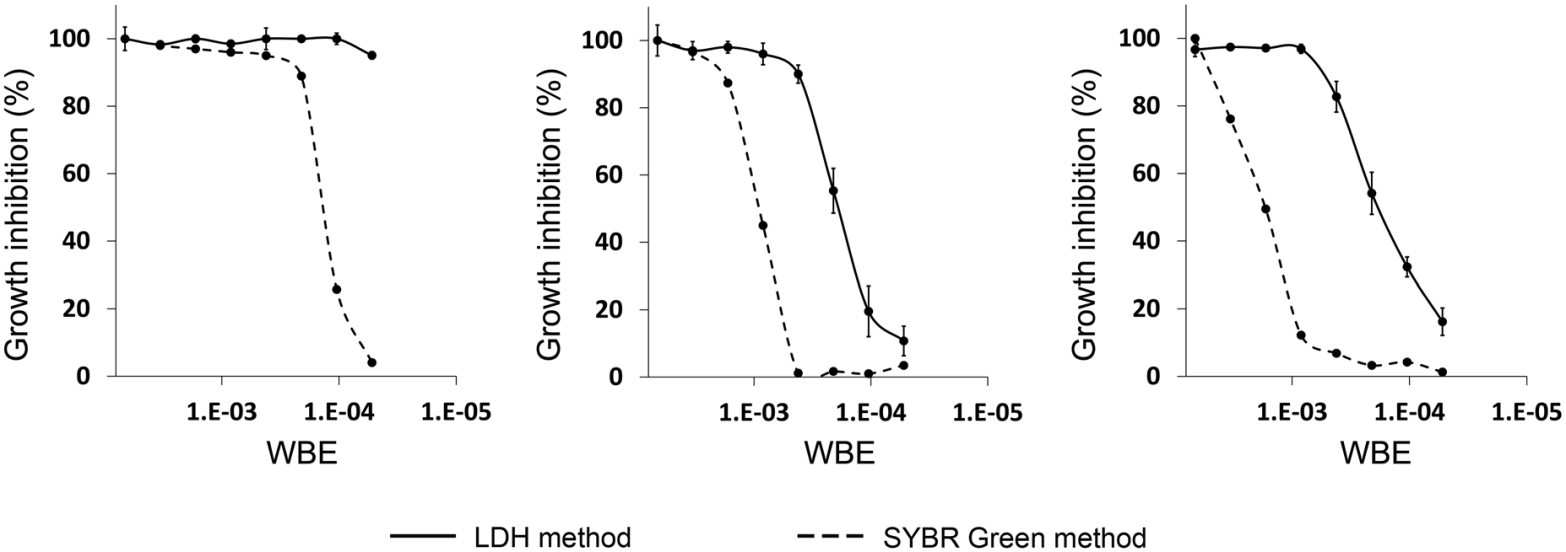
Examples of confirmation of the hits obtained in the primary assay. Both the LDH and SYBR Green assays were used for the confirmation of the positive extracts of the primary screening.

The MEDINA microbial natural product collection is composed of different modules. Initially, we focused our studies on a subset of 11,124 extracts from module A, containing both fungi and actinomycetes extracts in aqueous solution. A second subset of 8,560 extracts was selected from module B, containing fungal and bacterial extracts from previously selected strains known to produce antimicrobial activities. Extracts from this module contained 20% DMSO, and required a total evaporation followed by re-dissolving in methanol prior to the assay. Lastly, a third subset was selected from module C containing 560 extracts from bacteria of the classes Proteobacteria and Bacteroidetes in 20% DMSO. Since the assay dilution applied in this latter case was significantly high (1/3000), elimination of DMSO was not necessary (Table 1). Potential hits obtained in the primary screening were tested again in triplicate. We established that 70% of the hits obtained in the primary screen were confirmed to be active when assayed again in triplicate in an independent assay. The confirmed hits were then subjected to dose-response curves of at least 8 points obtained from serial dilutions of the extracts and using the PfLDH assay to establish potency. Extracts with a capacity to inhibit growth of at least 70% when diluted 8 times were then assayed in dose-response experiments with the secondary SYBR Green assay (Fig 2). While interference in fluorescent intensity assays in general by natural product extracts is well documented [27, 28], here the SYBR Green assay is used as a counter screen and it is unlikely that extract interference is an issue in both absorbance and fluorescence based assays. Therefore, all the extracts corroborating the inhibition in this assay were considered as confirmed positive hits of the primary screening. As new antimalarial compounds were sought, and in order to avoid the growth of extracts with known toxic compounds, all the confirmed hits obtained in the primary screening were subjected to LC-MS analysis, which allows the detection and identification of the main components of the extract.

**Fig 3:**
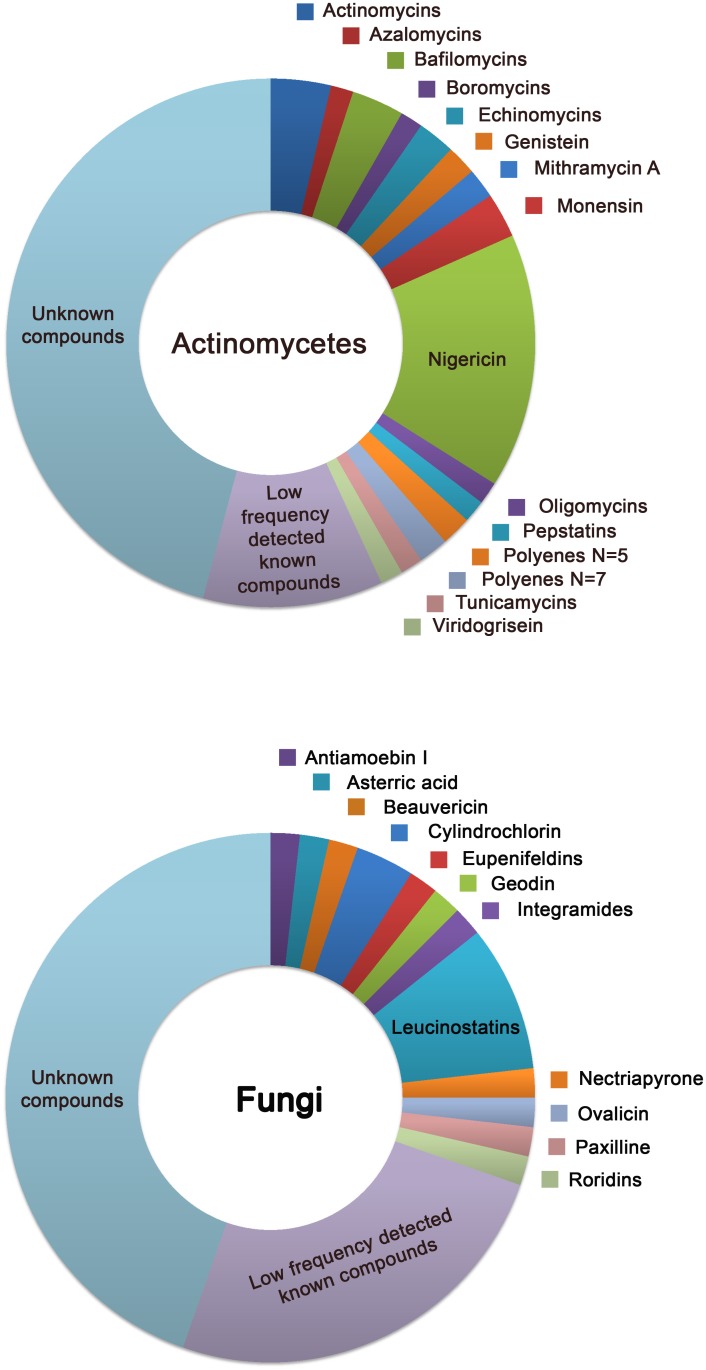
Compounds detected by LC-MS among the positive extracts of module A. The known products detected are presented depending on the microorganism origin of the extract (fungi or actinomycetes). The group of underrepresented compounds comprises all the compounds that are detected in only one extract.

The known compounds detected by LC-MS are shown in Fig 3. A database search was performed using an in-house developed application, which matches UV-LC-MS data of metabolites in the active extracts to UV-LC-MS data of known metabolites stored in our proprietary database obtained using the same LC-MS conditions [23]. The results are presented taking into account the source of the extract, actinomycetes or fungi. In the case of actinomycetes, antibiotics such as nigericins, oligomycins and actinomycins, among others, were identified. Nigericins are ionophores that catalyze the electroneutral exchange of K^+^ for H^+^ [29], whereas oligomycins and actinomycins act at the level of mitochondrial function and transcription, respectively [30, 31]. Many of the known compounds identified are produced by species of the genus *Streptomyces*. A total of 11% (actinomycetes) and 25% (fungi) of the hits correspond to known compounds that were detected at low frequency in the positive extracts. In the case of fungi, several known compounds such as the leucinostatins were identified, which have been shown to exert their cytotoxic action by perturbing mitochondrial oxidative phosphorylation [32]. Up to 45% of the hits correspond to extracts in which no known compounds were identified using MEDINA’s proprietary database.

After de-replication of extracts with known compounds, 60 extracts of module A, 150 of module B and 21 of module C were chosen for further characterization. Nineteen extracts from actinomycetes and 28 from fungi from module B were chosen for further scale-up according to the potency and the taxonomy of the producing strains. In the case of module C, since the data of cytotoxicity in three different human cell lines were available, only those extracts with no previously described cytotoxic effect were subjected to small scale growth, thereby reducing the number of extracts from 21 to 5.

### Small scale growth for bioassay-guided fractionation

After primary screening and hit selection, a critical step was the reproduction of the activity observed against *P*. *falciparum* in a new growth of the microorganism, assuring that the compound responsible for growth inhibition is present in the new extract. This confirmation was carried out in two phases, first a small scale growth (100 mL volume) was performed and, after confirmation of the bioactivity, a second large scale growth (1 L) was carried out to generate enough material for the isolation of the active molecule.

After small scale growth, the extracts generated were assayed in dose-response experiments to confirm inhibition of *Plasmodium* growth of more than 70%. Once the activity was confirmed, the positive samples were subjected to semipreparative HPLC fractionation, and the resulting fractions (80 fractions per sample) were analyzed for antiplasmodial activity. Plates were assayed at different concentrations to delimit the fractions containing the highest percentage of growth inhibition and, thus, identifying those that contain higher amounts of the most potent bioactive components. Once defined, the presence of known compounds was again established by LC-MS and LC-HRMS. After growth and fraction analysis, 17 extracts were further confirmed as containing potentially novel compounds and therefore selected for scale-up growth and isolation of the bioactive components.

### Growth scale-up and bioassay-guided purification

After verification of the antimalarial compound production in the small scale growth, the producing strain was cultivated in higher volumes (1 L), in order to provide enough sample for purifying the compounds responsible for the antimalarial activity. After this growth step, the extracts produced were first subjected to a low-resolution chromatographic step on SP-207ss resin generating 8–10 fractions, each of which was evaluated in a dose-response manner against *P*. *falciparum*. The most active fractions were then separated into several fractions by semipreparative or preparative HPLC, in a similar way to the procedure followed for the small scale regrowth. Fractions and subfractions were assayed for antiplasmodial activity and pure compounds responsible for the activity were re-purified from the most active fractions through semipreparative HPLC.

### Identification of pure compounds

**Fig 4:**
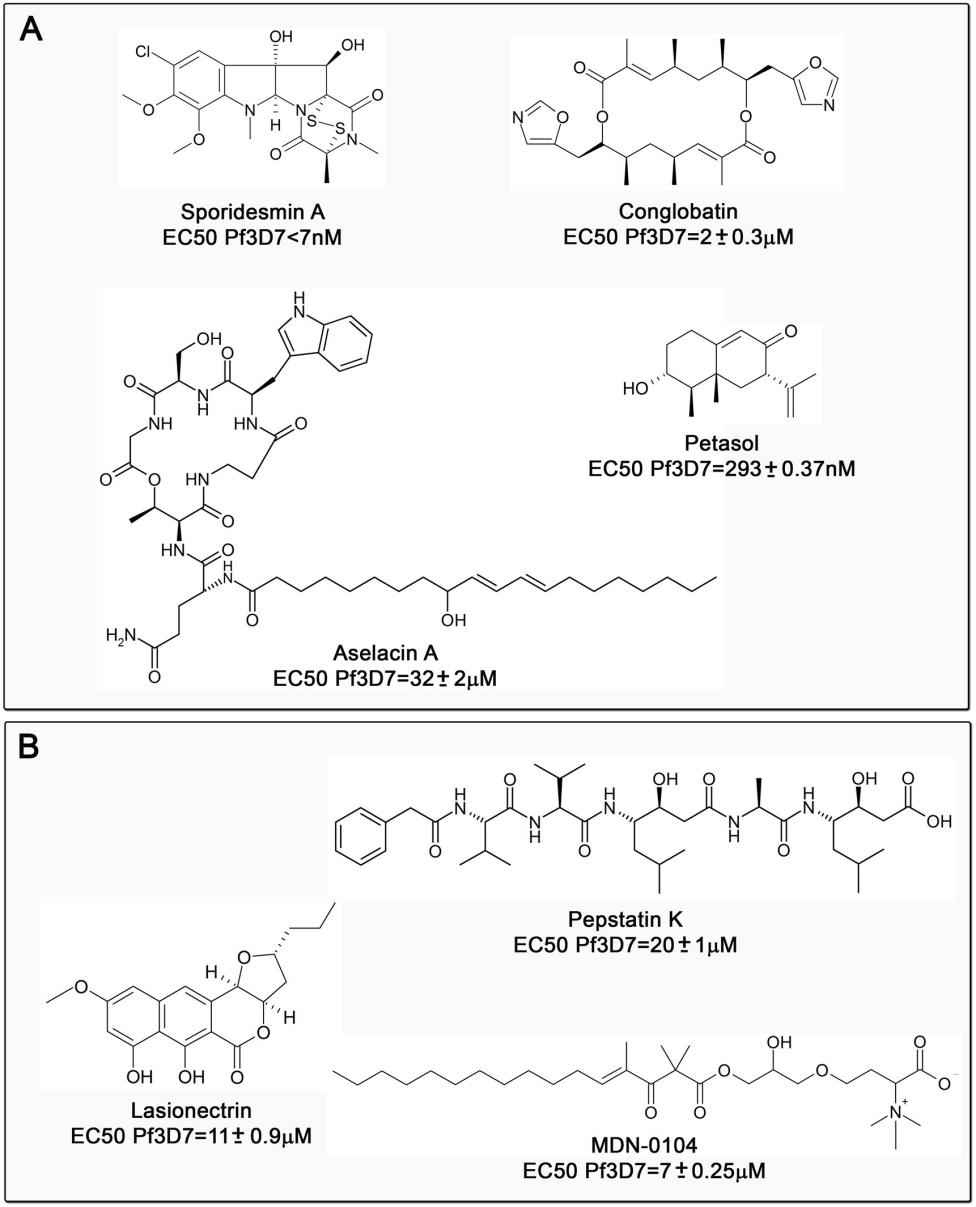
Compounds detected in the screen. Known (A) and unknown (B) compounds are indicated. The corresponding EC_50_ values, calculated using the LDH method, are shown below each compound. Results of mean and standard deviation for the EC_50_ values are from three independent experiments in triplicate.

Although not initially identified, final purification allowed for the identification of four known bioactive fungal compounds: aselacin A, petasol, sporidesmin A and conglobatin. In addition, three new compounds were identified. One is pepstatin K, a new peptide belonging to the pepstatin family containing two units of the unusual γ-amino acid statin ((3*S*,4*S*)-4-amino-3-hydroxy-6-methylheptanoic acid) produced by the *Kitasatopora mediocidica* F-136,264. On the other hand lasionectrin produced by *Lasionectria* sp. CF-176994, a naphtopyrone first discovered in this screening and which has been recently purified from fungal extracts [15] and a novel fungal betaine lipid MDN-0104 produced by Heterospora chenopolii CBS109836 were obtained [16]. We have previously reported the isolation and structures of these two latter compounds [15, 16]. Additionally two new cyclic peptides and a new tetramic acid are presently under evaluation and will be published elsewhere. The structure and EC_50_ obtained for each compound is indicated in Fig 4.

### Structural characterization of pepstatin K

**Fig 5:**
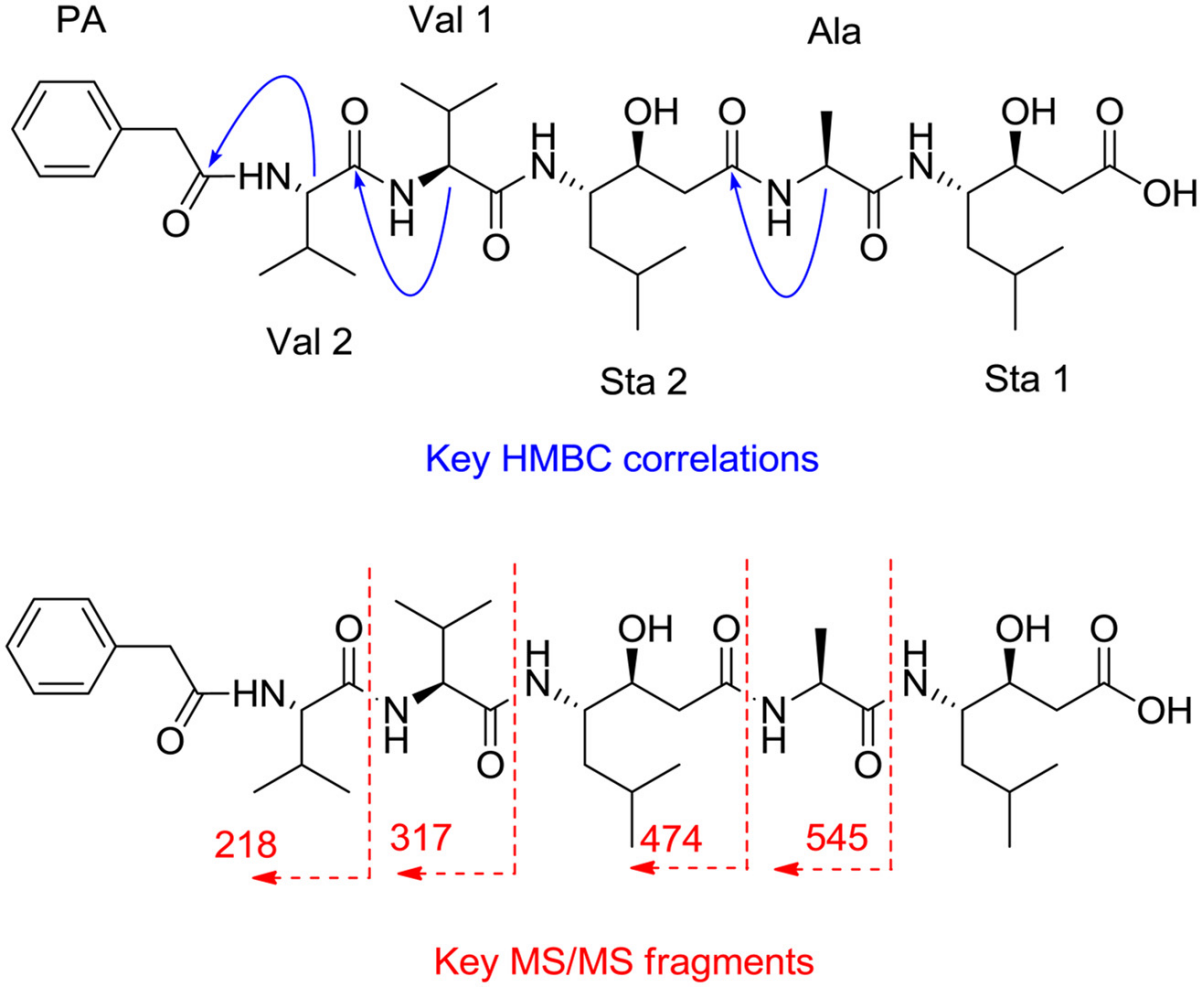
Key HMBC correlations (H to C) and MS/MS fragments observed in the spectra of pepstatin K.

A pseudomolecular ion at m/z 720.4555 obtained by ESI-TOF analysis established a molecular formula of C_37_H_61_N_5_O_9_ for pepstatin K (calc. for C_37_H_62_N_5_O_9_^+^ 720.4542). Analysis of the 1D (^1^H and ^13^C) and 2D (COSY, HSQC and HMBC) NMR spectra of the compound (Table 2) revealed the presence in the molecule of several amino acid units, including two valine, one alanine and two statine residues. Additionally, several signals in the low field region of the ^1^H NMR spectrum and the presence of six signals in the *sp*^*2*^ region of the ^13^C NMR spectrum accounted for the presence in the molecule of a phenyl containing residue that was eventually identified as 2-phenyletanoic acid (PE) based on correlations observed in the HMBC spectrum. The sequence of amino acid residues was partially established by analysis of key cross-peaks observed in the HMBC spectrum and MS/MS fragmentation (Fig 5). Thus, a correlation between the proton at δ_H_ 4.31 and a carbon at δ_C_ 173.8 was indicative of the partial sequence Sta 2-Ala. Additional correlations between proton signals at δ_H_ 4.10 and 4.18 to carbons at δ_C_ 173.9 and 174.2, respectively, confirmed the sequence PE-Val 2-Val 1. Additionally, the slight downfield shift of the carbonyl group of Sta 1 compared to that of Sta 2 (175.3 vs 173.8) determined the presence of a free carboxylic acid in Sta 1. The full sequence of the molecule was confirmed by key fragments observed in the MS/MS spectrum of the molecule where ions at *m/z* 218, 317, 474 and 545 were indicative of the breakage of the Val 2-Val 1, Va1 1-Sta 2, Sta 2-Ala, and Ala-Sta1 amide bonds, respectively. ^1^H NMR and ^13^C NMR spectra for pepstatin K are shown in S1 Appendix.

**Table 2:** NMR data for pepstatin K (500 MHz, CD_3_OD, at 24°C).

Amino acid	Position	δ_C_, mult	δ_H_, (mult, *J* in Hz)	COSY	HMBC
Sta 1	CO	175.3, C			
	*α*	39.9, CH_2_	2.44 (dd, 16.1, 4.4), 2.36 (m)	*β*	CO, C_*β*_, C_*γ*_
	*β*	70.9, CH	4.03 (m)	*α*, *γ*	CO, C_*α*_
	*γ*	52.5, CH	4.00 (m)	*β*, *δ*	
	*δ*	41.4, CH_2_	1.57 (m), 1.35 (m)	*γ*, *ε*	C_*γ*_, C_*ε*_, C_*ζ*_, C_*ζ’*_
	*ε*	25.9, CH	1.63 (m)	*δ*, *ζ*, *ζ’*	C_*δ*_, C_*ζ*_, C_*ζ’*_
	*ζ*	23.7, CH_3_	0.92 (m)	*ε*	C_*ε*_, C_*ζ*_
	*ζ’*	22.4, CH_3_	0.89 (m)	*ε*	C_*ε*_, C_*ζ’*_
Ala	CO	175.4, C			
	*α*	50.9, CH	4.31 (q, 7.3)	*β*	CO, C_*β*_, CO-Sta 2
	*β*	18.3, CH_3_	1.36 (d, 7.3)	*α*	CO, C_*α*_
Sta 2	CO	173.8, C			
	*α*	41.4, CH_2_	2.36 (m), 2.31 (dd, 14.7, 5.9)	*β*	CO, C_*β*_, C_*γ*_
	*β*	71.1, CH	4.01 (m)	*α*, *γ*	CO, C_*α*_
	*γ*	52.8, CH	3.90 (m)	*β*, *δ*	
	*δ*	41.2, CH_2_	1.57 (m), 1.35 (m)	*γ*, *ε*	C_*γ*_, C_*ε*_, C_*ζ*_, C_*ζ’*_
	*ε*	25.9, CH	1.63 (m)	*δ*, *ζ*, *ζ’*	C_*δ*_, C_*ζ*_, C_*ζ’*_
	*ζ*	23.7, CH_3_	0.92 (m)	*ε*	C_*ε*_, C_*ζ*_
	*ζ’*	22.4, CH_3_	0.89 (m)	*ε*	C_*ε*_, C_*ζ’*_
Val 1	CO	173.8, C			
	*α*	61.0, CH	4.10 (d, 7.9)	*β*	CO, CO-Val 2, C_*β*_, C_*γ*_, C_*γ’*_
	*β*	31.5, CH	2.05 (m)	*α*, *γ*, *γ’*	CO, C_*α*_, C_*γ*_, C_*γ’*_
	*γ*	18.9, CH_3_	0.93 (m)	*β*	C_*α*_, C_*β*_, C_*γ’*_
	*γ’*	20.0, CH_3_	0.95 (m)	*β*	C_*α*_, C_*β*_, C_*γ*_
Val 2	CO	173.9, C			
	*α*	60.7, CH	4.18 (d, 7.9)	*β*	CO, CO-Ar, C_*β*_, C_*γ*_, C_*γ’*_
	*β*	31.7, CH	2.05 (m)	*α*, *γ*, *γ’*	CO, C_*α*_, C_*γ*_, C_*γ’*_
	*γ*	18.9, CH_3_	0.93 (m)	*β*	C_*α*_, C_*β*_, C_*γ’*_
	*γ’*	19.9, CH_3_	0.95 (m)	*β*	C_*α*_, C_*β*_, C_*γ*_
PE	CO	174.2, C			
	CH_2_-Ar	43.6, CH_2_	3.59 (m)	*α*	CO, Ar-C, C_*α*_
	Ar-C	136.9, C			
	CH-*α*, *α’*	130.2, CH	7.30 (m)	CH_2_, *γ*	CH_2_, C_*γ*_
	CH-*β*, *β’*	129.6, CH	7.30 (m)	*γ*	Ar-C
	CH-*γ*, *γ*’	127.9, CH	7.23 (m)	*α*, *β*	C_*β*_

## Discussion

The screening described herein has proved to be a valuable and exploitable tool in the search for new antimalarials. Due to the growing need for new drugs to treat this disease, exploring new sources of chemical scaffolds could contribute to the identification of novel drugs with new modes of action. Chemical libraries created by iterative synthesis around a few families of compounds are not always the best option when the objective is to identify non-previously exploited novel scaffolds. While several high throughput screens against *P*. *falciparum* of vast compound collections have been previously published [18, 26], to date no high throughput studies have been performed in the field of microbial natural products. One of the potential challenges of using natural products collections is the expertise required to address the complexity of isolating and elucidating the pure compound responsible for the activity from positive extract identified in a primary screen. Unfortunately this process sometimes involves the identification of previously described compounds as is the case of petasol [33] obtained in the present study. The application of this sensitive technology [18] combined with the use of semi-automated fractionation and miniaturized LC-MS, LC-HRMS and NMR analyses, has allowed for a fast and efficient identification of minor extract components at an early stage of the discovery process thus optimizing the identification of novel actives. Likewise, high throughput technology has permitted the analysis of hundreds of extracts and multiple fractions in a highly cost-effective and time-efficient manner. Another limitation for natural product exploitation is in some cases the low yield of the active compounds that can be overcome applying different scale-up strategies as well as traditional strain optimization of the production of distinct metabolites or heterologous expression in suitable hosts.

While the identification of novel compounds is of major interest, known compounds should not be disregarded since in many cases their antimalarial activity has not been previously described and they could thus provide a starting point for the development of new antiplasmodial agents. Drug repurposing constitutes a productive avenue for the identification of new therapies. Examples are the use of antifungal imidazoles recently tested in clinical trials against Chagas disease [34] or the recently approved combination therapy of nifurtimox (an antichagasic agent) with eflornithine for the treatment of sleeping sickness [35].

In summary, the compounds identified in the present natural products screening provide valuable information for antimalarial drug discovery from microbial sources. Although the most druggable compounds identified exhibit EC_50_ values in the micromolar range, these hits could be considered as good starting points for a lead optimization process. While the present report describes the first data obtained in the screening of approximately one-seventh of the extracts available, efforts are currently underway to complete the analysis of the wealth of information contained within the whole MEDINA natural product extract collection.

## Supporting Information

S1 Appendix ^1^H NMR and ^13^C NMR spectra for pepstatin K. (PDF)

S1 Fig Determination of chloroquine EC_50_ in *P*. *falciparum* by the LDH assay. (TIF)
