# Supplementary material for: Discovery of New Compounds Active against Plasmodium falciparum by High Throughput Screening of Microbial Natural Products
Source: PLoS One. 2016 Jan 6;11(1):e0145812. doi: 10.1371/journal.pone.0145812 (PMC4703298; doi:10.1371/journal.pone.0145812)
Supplement: S1 Appendix — (PDF) [file pone.0145812.s001.pdf]

Appendix S1

^{1}H NMR and ^{13}C NMR spectra for pepstatin K

Discovery of New Compounds Active against *Plasmodium falciparum* by High Throughput Screening of Microbial Natural Products

Guiomar Pérez-Moreno[1], Juan Cantizani[2], Paula Sánchez-Carrasco[1], Luis Miguel Ruiz-Pérez[1], Jesús Martín[2], Noureddine el Aouad[2], Ignacio Pérez-Victoria[2], José Rubén Tormo[2], Víctor González-Menendez[2], Ignacio González[2], Nuria de Pedro[2], Fernando Reyes[2], Olga Genilloud[2], Francisca Vicente[2] and Dolores González-Pacanowska[1]*

1 Instituto de Parasitología y Biomedicina "López-Neyra". Consejo Superior de Investigaciones Científicas. Parque Tecnológico de Ciencias de la Salud, Avenida del Conocimiento, s/n. 18016-Armilla (Granada), Spain.

2 Fundación MEDINA. Parque Tecnológico de Ciencias de la Salud, Avenida del Conocimiento, 34.18016-Armilla (Granada), Spain.

*Author to whom correspondence should be addressed. Tel: +34 958181631. Fax: +34 958181632. E-mail: dgonzalez @ipb.csic.es

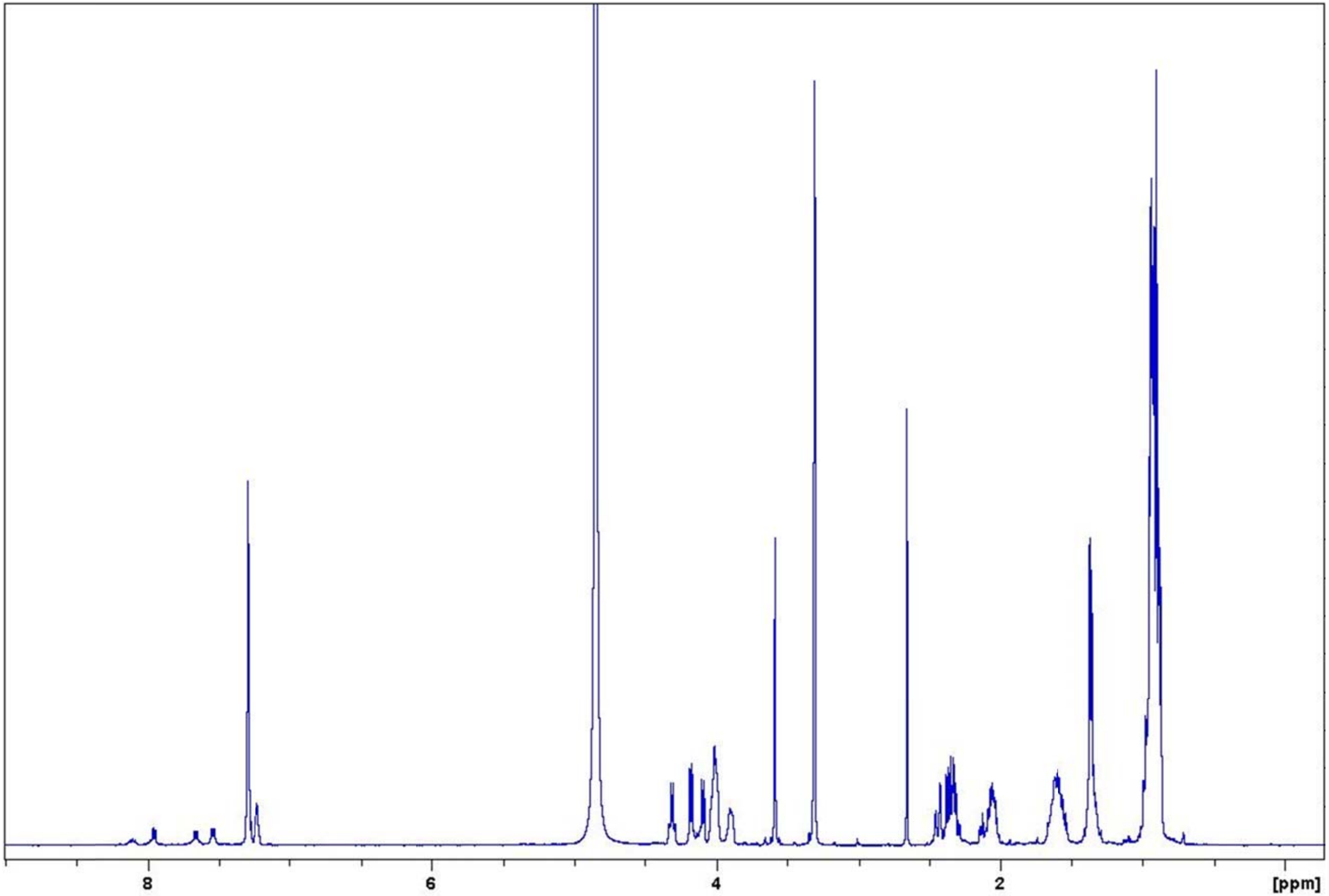

^{1}H NMR spectrum of pepstatin K (500 MHz, CD_3OD at 24 ºC)

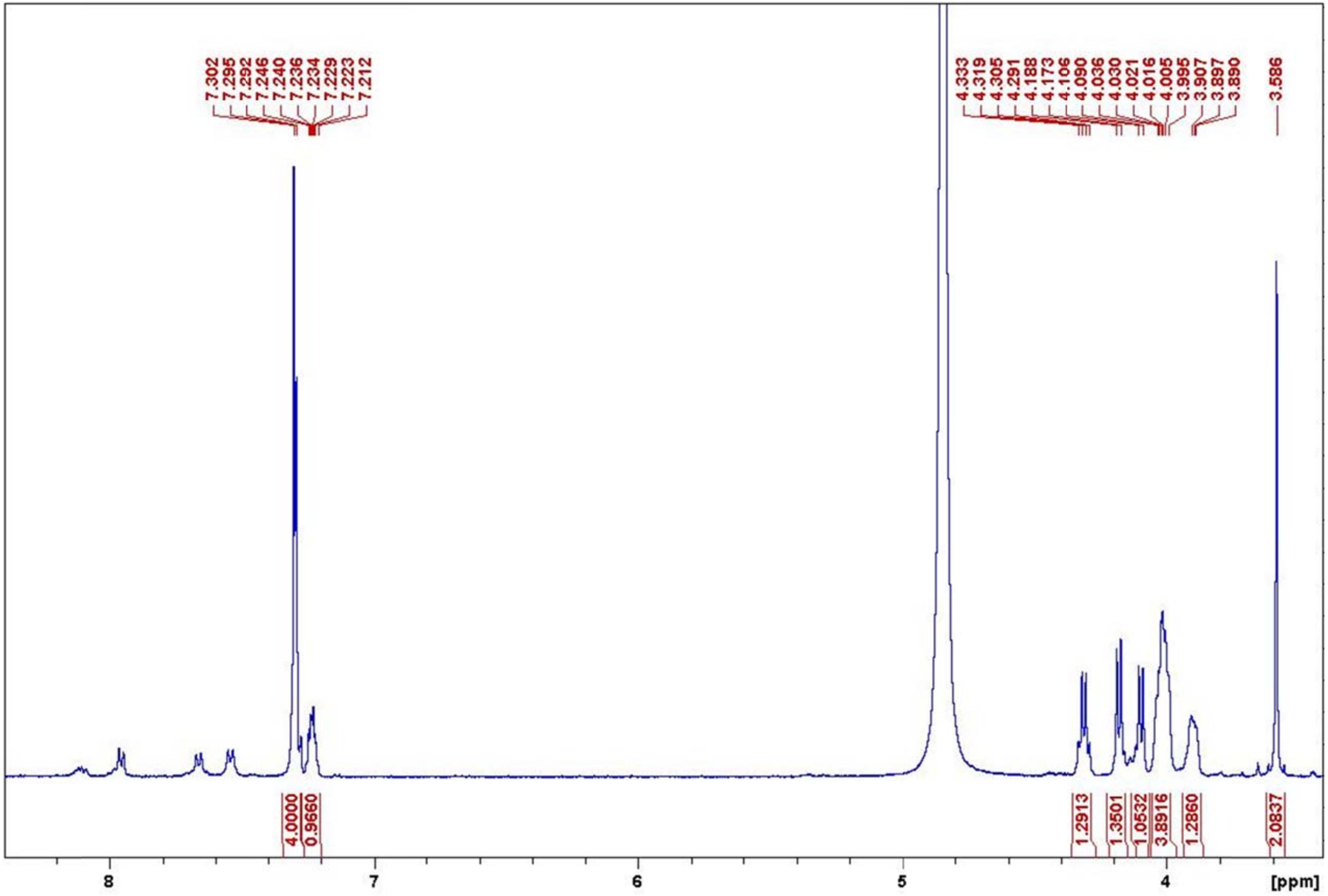

Expansion of the ^{1}H NMR spectrum of pepstatin K (500 MHz, CD_3OD at 24 ºC)

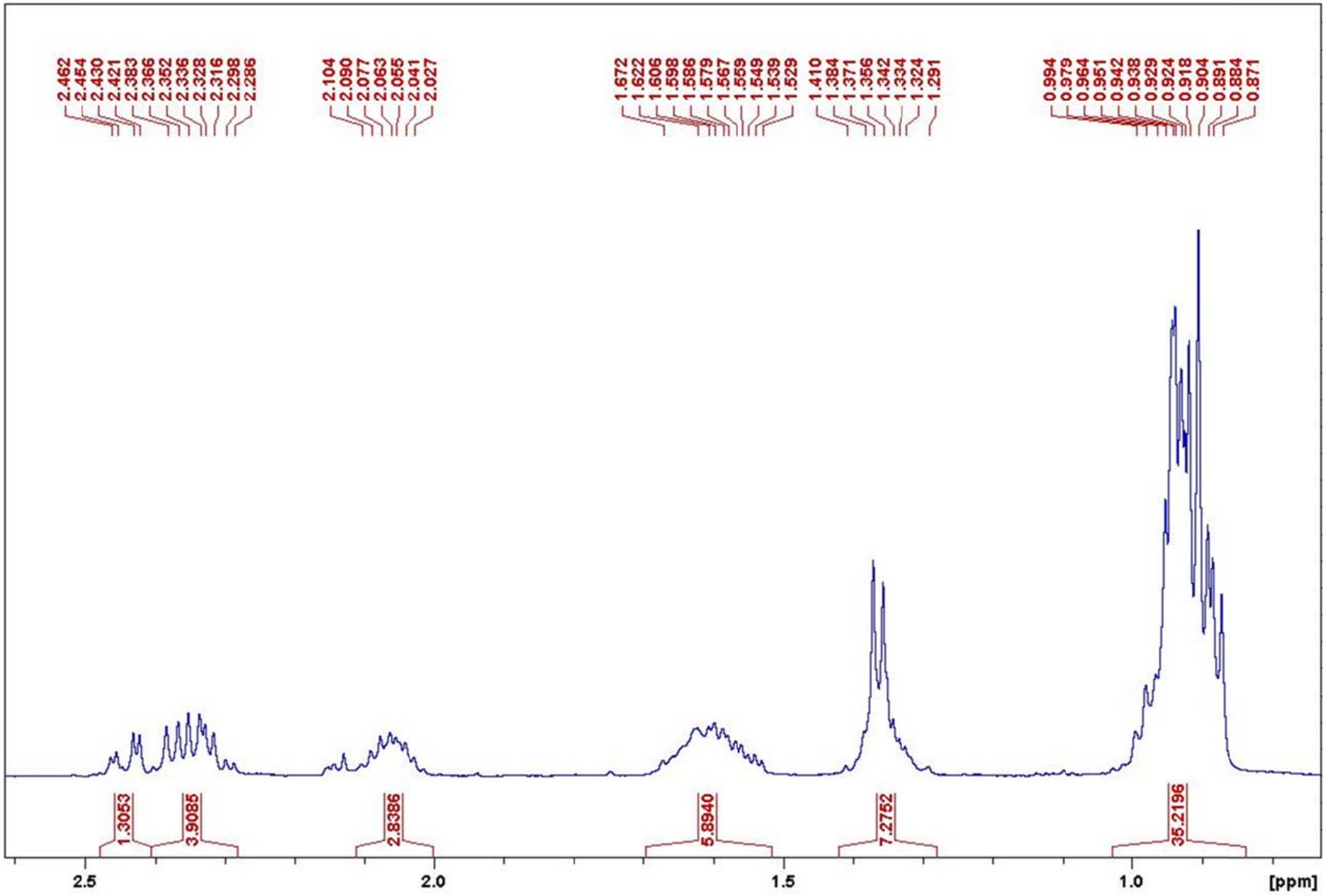

Expansion of the ^{1}H NMR spectrum of pepstatin K (500 MHz, CD_3OD at 24 ºC)

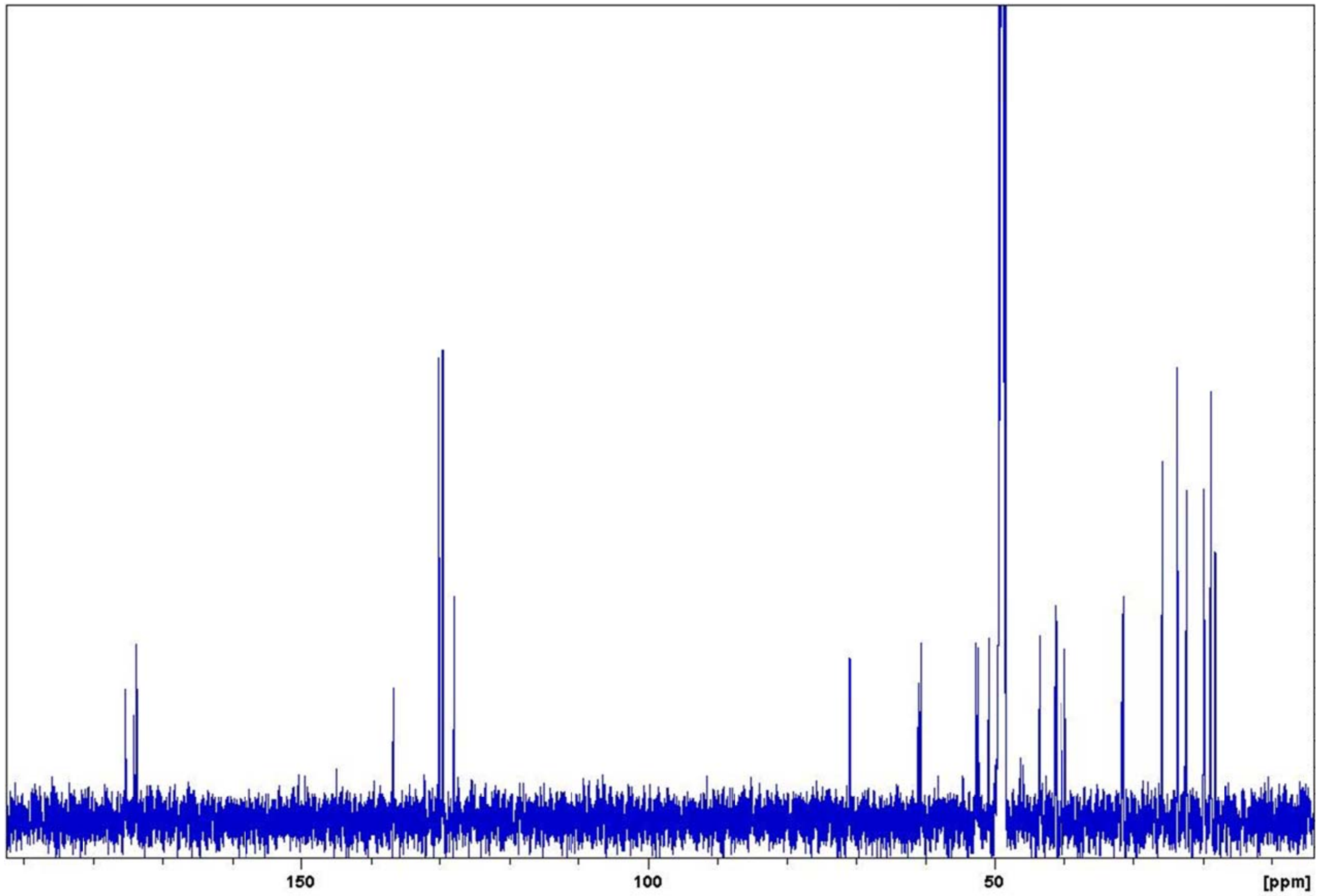

^{13}C NMR spectrum of pepstatin K (125 MHz, CD_3OD at 24 ºC)

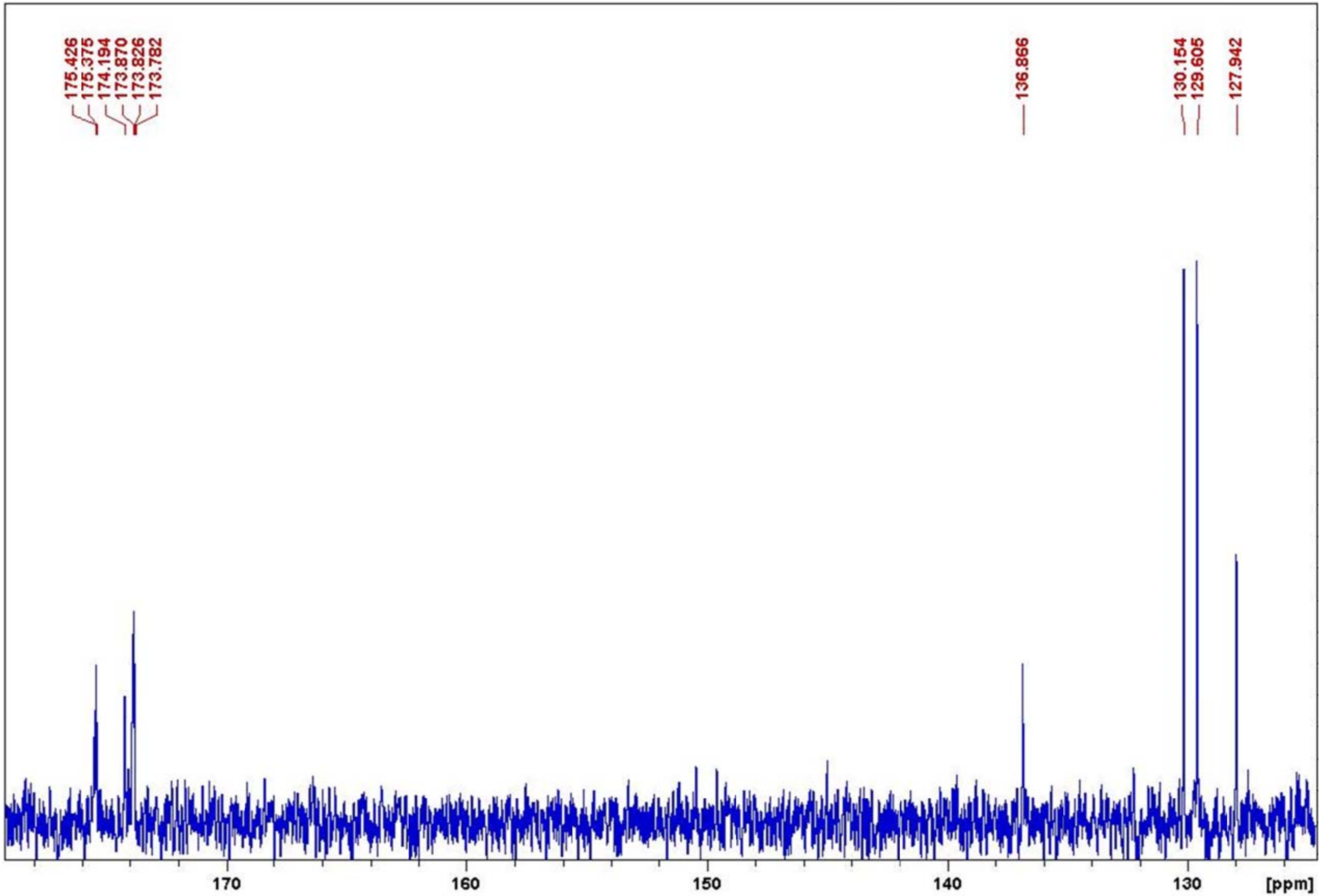

Expansion of the ^{13}C NMR spectrum of pepstatin K (125 MHz, CD_3OD at 24 ºC)

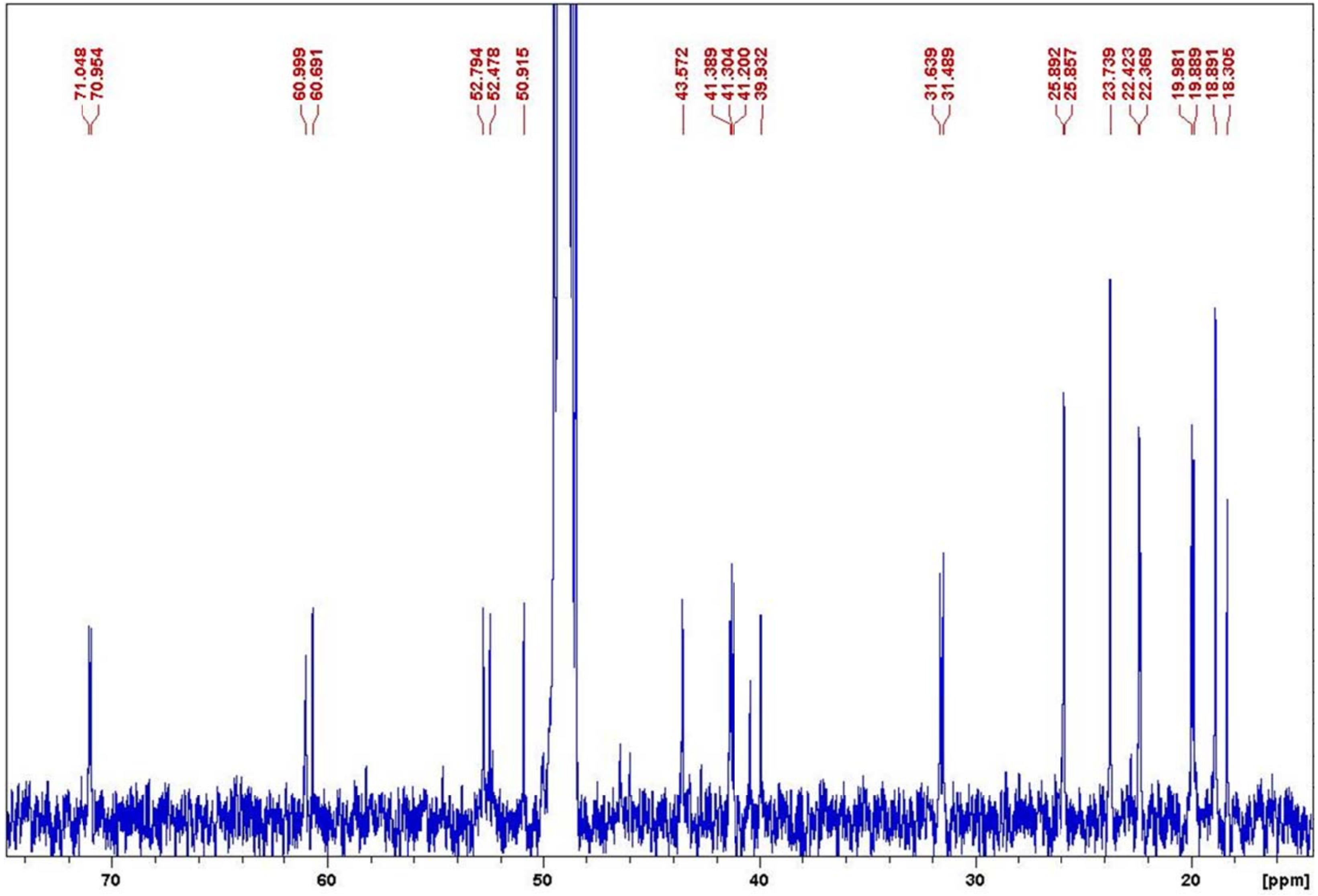

Expansion of the ^{13}C NMR spectrum of pepstatin K (125 MHz, CD_3OD at 24 ºC)

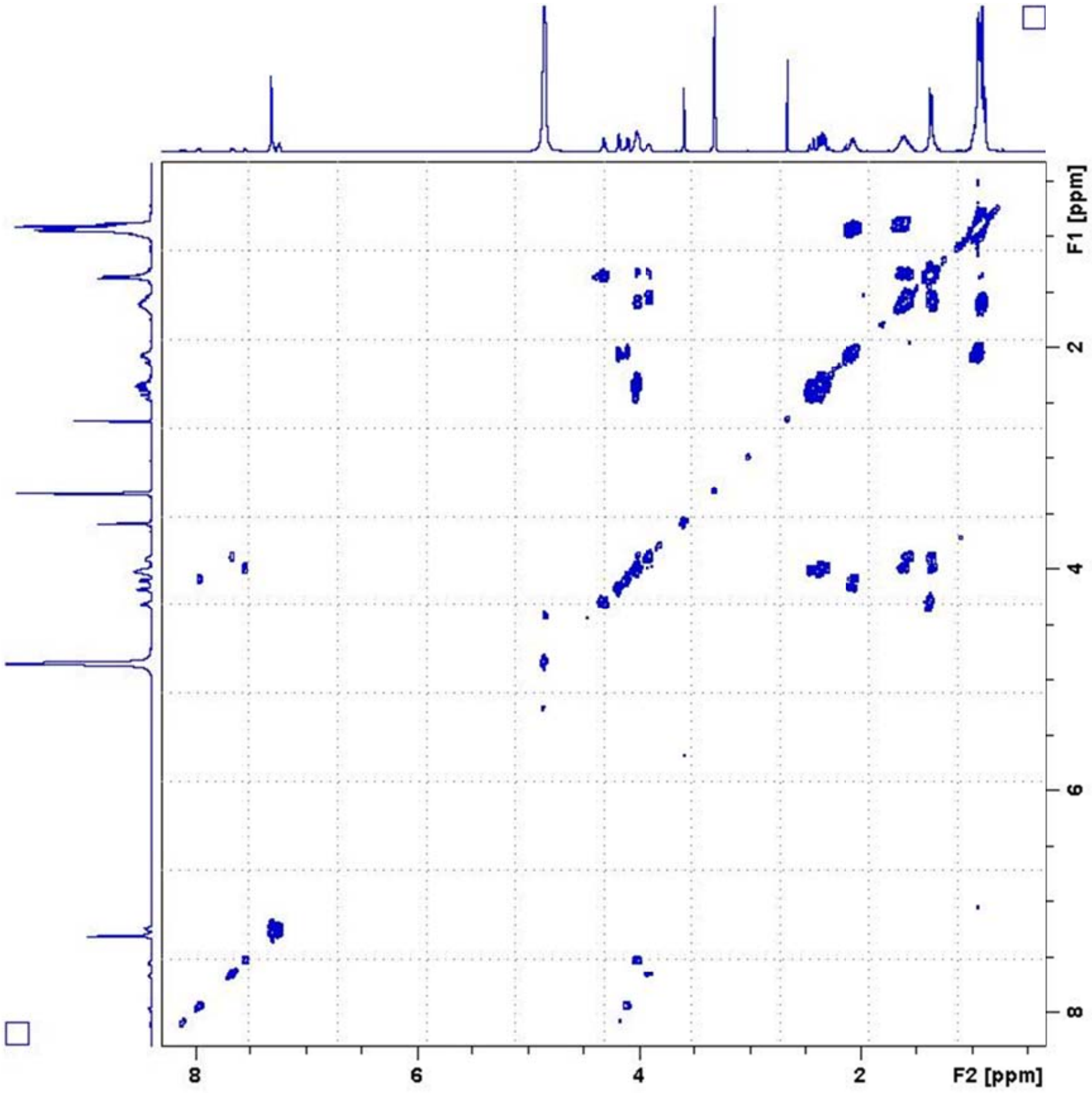

COSY spectrum of pepstatin K

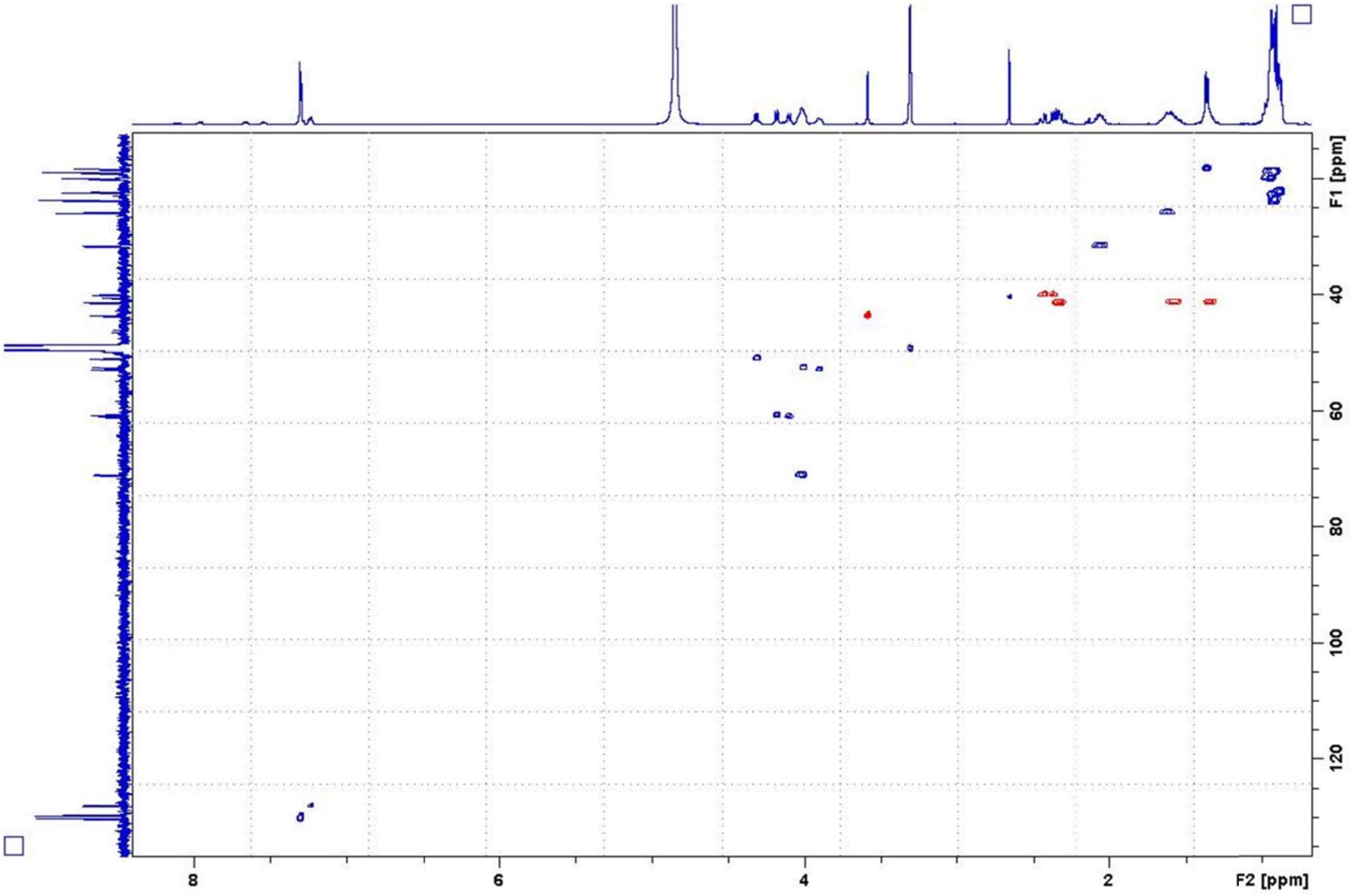

HSQC spectrum of pepstatin K

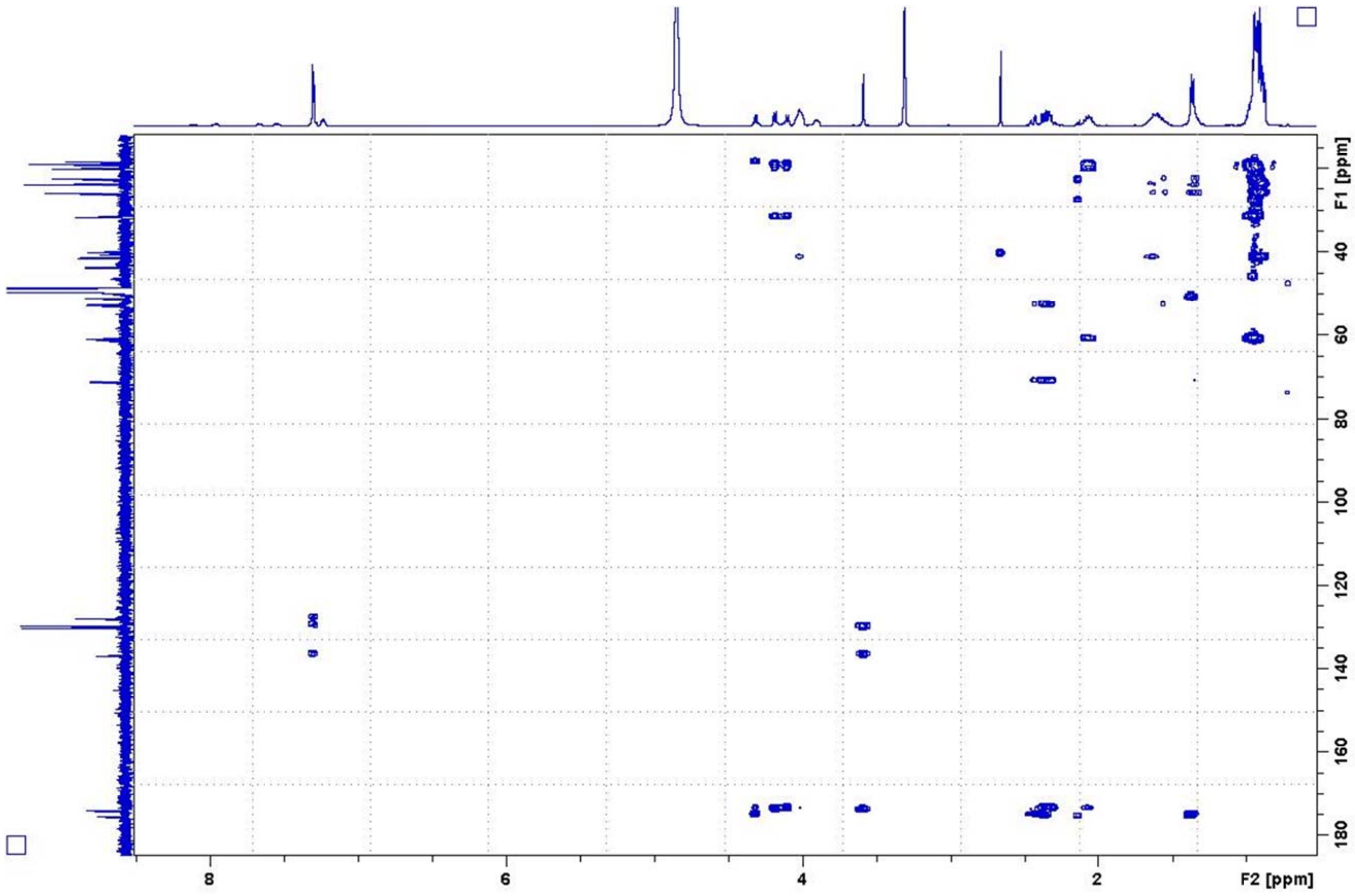

HMBC spectrum of pepstatin K

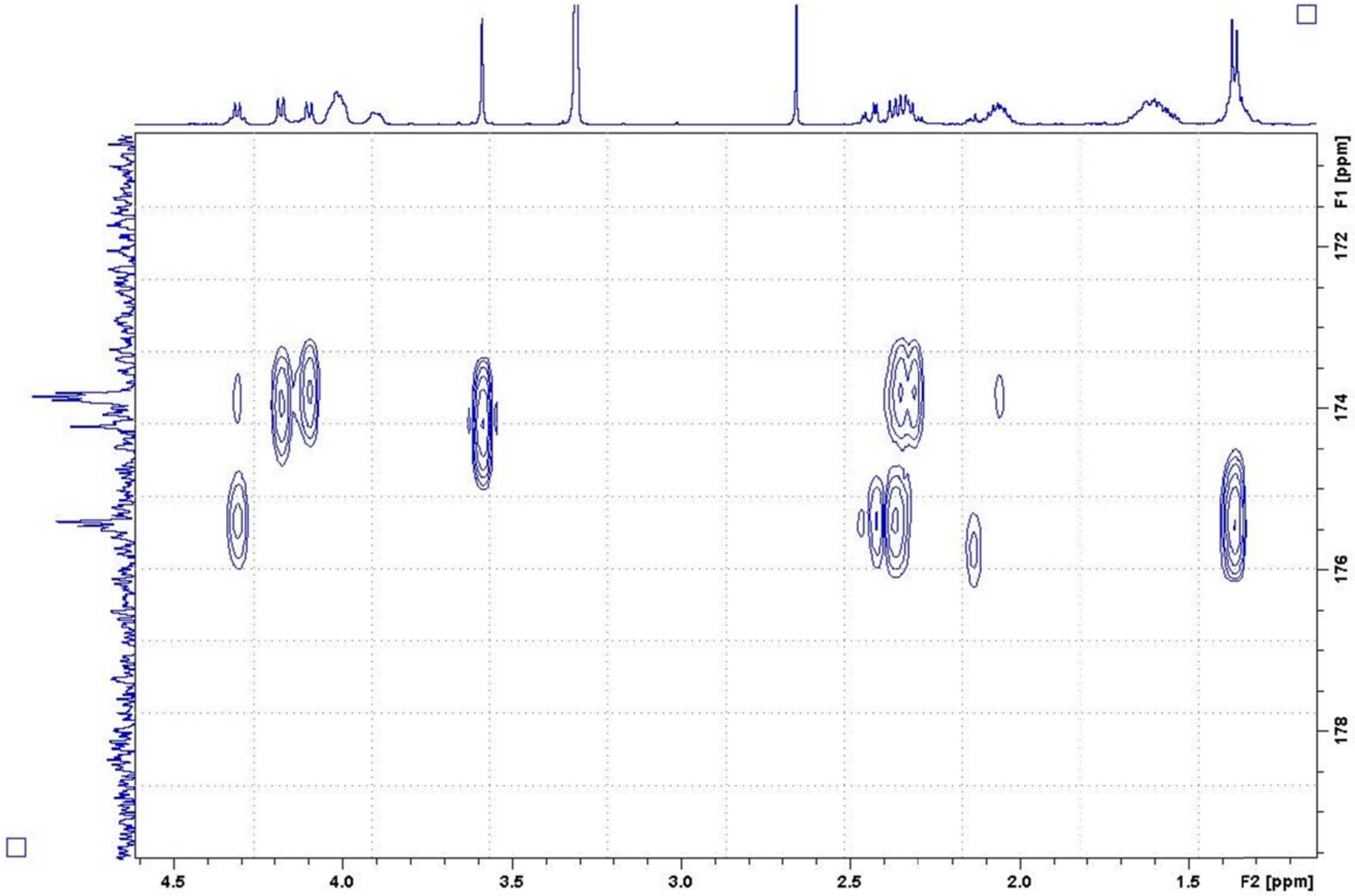

Expansion of the HMBC spectrum of pepstatin K showing the carbonyl region
