# Supplementary figures and images for: Discovery of New Compounds Active against Plasmodium falciparum by High Throughput Screening of Microbial Natural Products
Source: PLoS One. 2016 Jan 6;11(1):e0145812. doi: 10.1371/journal.pone.0145812 (PMC4703298; doi:10.1371/journal.pone.0145812)

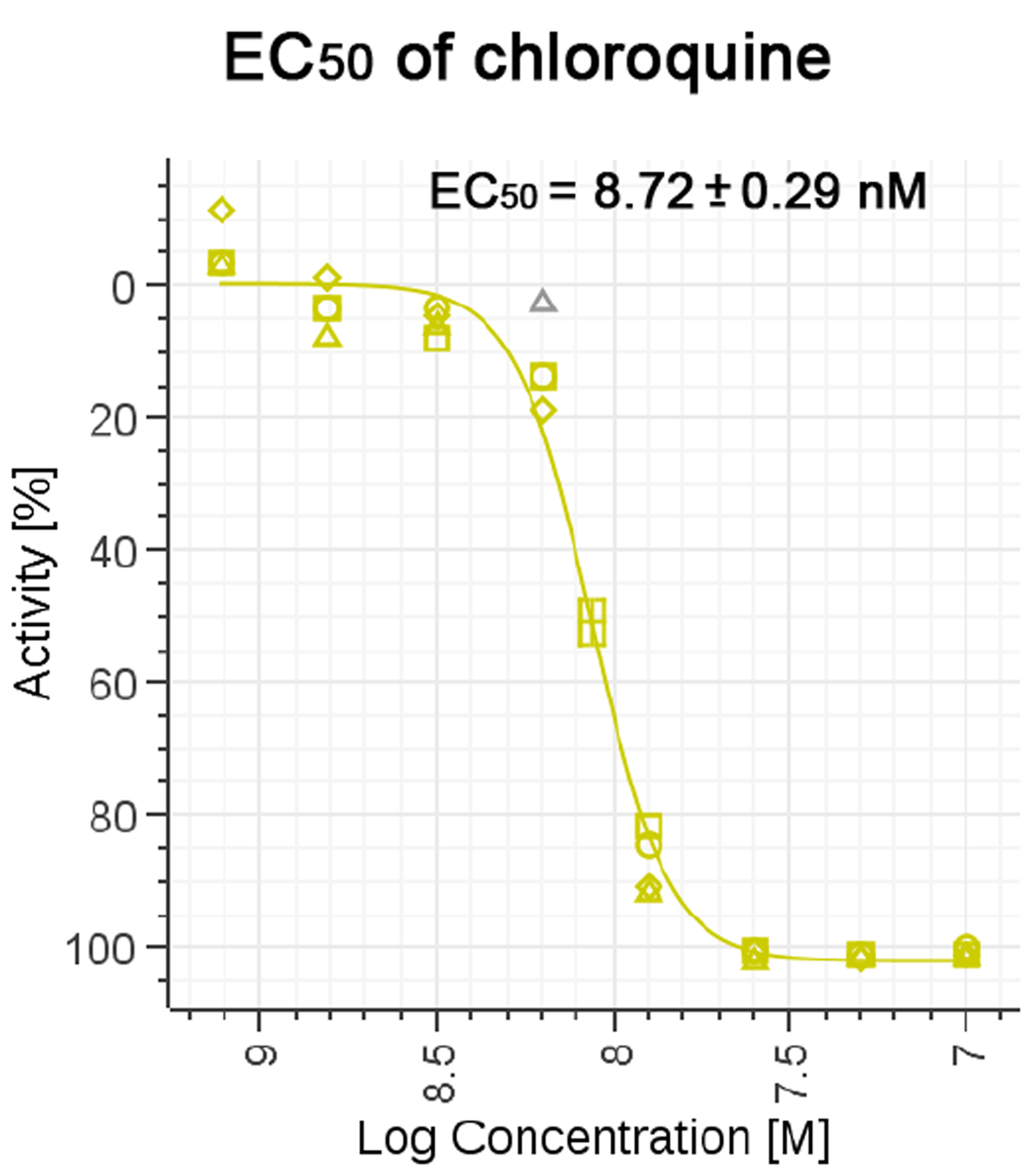

Supplement: S1 Fig — (TIF) [file pone.0145812.s002.tif]
